# Microseconds Matter

**DOI:** 10.1371/journal.pbio.1000405

**Published:** 2010-06-29

**Authors:** Catherine E. Carr, Katrina M. MacLeod

**Affiliations:** Department of Biology, University of Maryland, College Park, Maryland, United States of America

## Abstract

This Primer focuses on detection of the small interaural time differences that underlie sound localization.

Animals constantly detect and encode the location of sound sources in the environment. In order to determine the location of sounds in the horizontal plane, or azimuth, the auditory system employs the interaural time differences (ITDs) that arise when sound reaches one ear before the other. Given the tiny time differences involved, animals are remarkably accurate at localizing sounds. The range of time differences that are useful to the animal depends on the head size—for humans, it's about 600 µs, for gerbils about 150 µs. Humans and barn owls are both localization champions, with an ability to resolve sounds about 2° apart [1]. The task is easier for humans than for barn owls, because our heads are bigger (we have more microseconds per degree of azimuth), but all localizing animals detect time differences on the order of tens of microseconds. This temporal accuracy is remarkable, especially considering that individual neurons fire action potentials that can last a millisecond or more in duration.

ITDs are detected by specialized neurons that act as coincidence detectors in an area of the brainstem called the medial superior olive. The name reflects how these neurons work—they respond most reliably when they receive precisely synchronized, essentially simultaneous inputs from each ear. For coincidence detection to be useful in detecting very small time differences, the incoming sound information must first be encoded very precisely in the periphery, such that neurons fire action potentials in phase with the sound on a cycle-by-cycle basis. The phase-locked activity of the auditory nerve fibers in the periphery are relayed by the cochlear nucleus specialized for timing. The nervous system throws everything it's got at both making and keeping a temporally precise signal, from fast synapses to short sharp responses [2]. Precise, phase-locked inputs from the left and right cochlear nuclei converge on the coincidence detector neurons. These neurons respond maximally when inputs from the two sides coincide (or almost coincide), and minimally at an unfavorable ITD. When pure tones are used, the tuning curves have multiple peaks 2pi apart, revealing their dependence on interaural phase differences.

## Coding ITDs

In 1948, Jeffress proposed a circuit for detection of interaural time differences [3]. His circuit consists of two elements—delay lines and coincidence detectors—and elegantly explains both the measurement and the encoding of ITDs. The delay lines are created from variations in axonal path lengths, and the coincidence detectors are units that respond most vigorously when they receive inputs simultaneously from the axons converging from each ear [4]. This can only occur when the external time difference is exactly compensated for by the delay introduced by the axonal travel time.

Jeffress envisaged arrays of ITD detectors for each frequency band, each tuned to a different preferred ITD, so that the whole array could form a place map, also called a “labeled line” population code [1], and where the peak or maximum firing rate encoded ITD. However, for most low-frequency neurons, the ITD tuning curves are too broad, i.e. their peaks are too blunt, to make such an arrangement efficient. Consequently, from an “optimal coding” perspective, the peaks of the ITD tuning curves should not be as relevant as the slope of the curves [5],[6]. Furthermore, the peaks of the ITD curves often fall outside the range of ITDs available to the animal. Only when ITD tuning curves are relatively sharper, i.e. for neurons tuned to higher frequencies in animals with large ITD ranges, does a Jeffress-like arrangement become computationally efficient.

A landmark paper from McAlpine, Jiang, and Palmer (2001) focused on the relevance of the slope of the ITD curve to localization, and led to reexamination of ITD coding, especially a reevaluation of the Jeffress model's utility as a description of ITD coding [7]. The Jeffress model works well for birds, where delay lines create maps of ITD, even at low frequency sounds [8],[9]. For mammals with small heads, like guinea pigs and gerbils, the data do not fit the Jeffress model. Instead, small-headed mammals are hypothesized to use the “slope” of the ITD curve, or the change in firing rate. They could then estimate ITD by comparing the output of left and right coincidence detectors [7].

It's not often that neurobiologists are able to generate such explicit hypotheses about neural coding, and there has been a great deal of excitement and discussion about how ITDs are detected and which of the various coding strategies are used. The slope and peak solutions for encoding ITDs are not inconsistent, since both depend upon coincidence detection and convey ITDs to the midbrain through the distribution of firing rates across the population of neurons. In the best studied examples, barn owls appear to use the information in both peaks and slopes of the tuning curves [10],[11], while gerbils might use the “slope” code [7]. A more recent theoretical re-examination of the slope-peak paradox reveals that the presence of noise could affect coding choice. In low-noise environments, from an information theoretic point of view, it is advantageous to use a slope code, to obtain better discrimination between similar orientations [11]. In high-noise environments, however, it's better to operate near the maximal firing regime or peak. As pointed out by Solla [12], this “suggests the potential existence of an adaptive readout mechanism that would adjust its strategy according to the noise level.”

## Coarse and Fine Control of Delays

**Figure 1:**
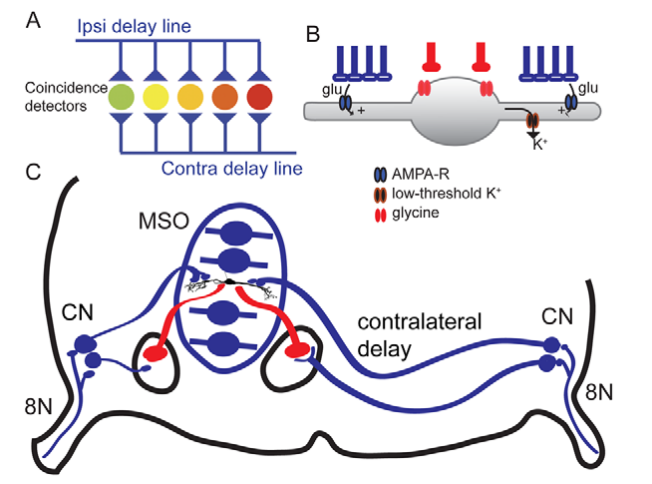
(A) The Jeffress model for the computation of ITDs. Monaural channels act as delay lines and project to an array of coincidence detectors that each tap the signal at a different ITD. The coincidence detectors are maximally active when the internal (axonal) delay is equal but opposite to the acoustic ITD. Thus the delay lines create a map of ITD, transforming the temporal code into a place code. (B) Dendritic computation enhances coincidence detection; ipsilateral and contralateral inputs are segregated onto different dendrites, allowing for shunting of out of phase postsynaptic current via a critically important potassium channel conductance with a low activation threshold (G_KLT_). G_KLT_ is densest near the cell body, greatly improves the time resolution of excitatory summation, and accelerates membrane repolarization [24]. (C) Neurons in the medial superior olive (MSO) encode interaural time differences by integrating bilateral excitatory inputs from both cochlear nuclei (CN) and bilateral inhibitory inputs from the lateral and medial nuclei of the trapezoid body (red). There is longer path length from the contralateral CN to the MSO. The MSO neurons are shown as schematics except for a single gerbil MSO neuron (modified from [29]).

The original Jeffress model showed equal path length delay lines converging on an array of coincidence detectors (Figure 1A). But studies in birds and mammals show a much longer path length to the coincidence detectors from the contralateral side than from the ipsilateral side (Figure 1C), introducing a delay line such that when sound reaches the contralateral ear first, the information travels along the longer contralateral axon to arrive simultaneously with inputs due to sound that reaches the ipsilateral ear. The nature of delay lines has been examined quite carefully in owls and chickens, and gross differences in axon length appear to be compensated for by differences in signal propagation times along each axon [13],[14]. The axons show differences in diameter and internodal distance, and Seidl et al. (2010) have also proposed that “Variations of parameters such as axon diameter, internode distance, and others [15] in the mammalian brainstem might be responsible for precise adjustments of physiological delays, thereby creating the framework and adjustments of the ITD detection circuit.”

New computer-aided reconstructions of axonal connections suggest that the observed path lengths cannot account for the distribution of best delays in the cat [16],[17],[18],[19]. Karino et al. (2010) suggest that some other mechanism(s) must contribute to the internal delays [18]. The principal contender for a biophysical mechanism to modify internal delays has been the timing and or magnitude of inhibitory input to the coincidence detectors [19],[20]. However, in the current issue of *PLoS Biology*, Jercog et al. present a novel mechanism to compensate for the robust delay caused by the longer contralateral path length [21].

The Jercog et al. data showed that there are asymmetries in the synaptic inputs to the coincident detector neurons such that the contralateral compound postsynaptic potential had a slower rise time than the ipsilateral postsynaptic potential. Why? Either there is an intrinsic biophysical difference between the two sets of dendrites (the structures receiving the signals), or there is more variation in the arrival times of the contralateral inputs. The second is more plausible, principally because Jercog et al. stimulate the ipsilateral inputs relatively close to the recording site, while the contralateral stimulating electrode is further away. Thus, any intrinsic variability in conduction velocity in the contralateral input bundle would be magnified. The authors performed their experiments in gerbil brain slices that preserved connectivity between the coincidence detector (the medial superior olive) and its inputs from the two cochlear nuclei (Figure 1). They observed an almost 500 µs conduction time difference between ipsi- and contralateral stimulation of cochlear nuclei recorded at 32°C. Of course, *in vivo*, this difference would not be so large. With a brain temperature near 38°C and a Q_10_ of 1.8 [22], an estimated conduction velocity in the slice of 4.9 m/s would yield an *in vivo* conduction velocity closer to 7 m/s and a shorter delay between ipsilateral and contralateral inputs.

These measurements made by Jercog and colleagues go a long way toward explaining the measured distributions of best ITDs in gerbils [15],[23]. The mean best ITD is found when the contralateral inputs lag ipsilateral inputs by about 135 µs, with characteristic delays ranging from 0–500 µs [15]. Perhaps the contralateral delays provide a “coarse” regulation of delay, shifting the coincidence window into the contralateral hemifield, and then synaptic events provide “fine” tuning. Jercog et al.'s paper in this issue, and other recent papers [24], certainly support a role for synaptic regulation of delay.

## Synaptic Events and Precise Regulation of ITDs

Most neurons respond best when their inputs arrive simultaneously, because of spatial and temporal summation. What sets ITD coincidence detection apart is the narrowness of the summation window and the exclusion of nonsynchronous inputs. True coincidence detectors also require more than simple summation—they should fire when inputs from two ears coincide, but not when two inputs from the same ear coincide. A neuron that sums its inputs linearly would not be able to distinguish between these two scenarios. The neurons in the ITD circuit meet this criterion: the minimal firing rate (in the trough of the tuning curve) that occurs during out of phase binaural stimulation is actually *less* than the monaural firing rates. Thus coincidence detectors behave more like biophysical AND-gates than simple summation devices. Several mechanisms contribute to this effect, including the segregation of the ipsilateral and contralateral inputs onto different dendrites and the shunting of the postsynaptic current via a critically important potassium channel conductance with a low activation threshold (G_KLT_) [24],[25]. Low threshold potassium channels are crucial multitaskers in the coincidence detectors. G_KLT_ activates with only small depolarization [26] and sets the time constant of the membrane by reducing the membrane resistance to unusually low values. Because of the G_KLT_, coincidence detectors typically have very low input resistances and thus very rapid responses to changes in voltage (τ_m_ of 0.3–1.5 ms). Spike triggering is very sensitive to the rate of rise of the voltage in these and other coincidence detector neurons [27],[28].

Together, synaptic and intrinsic mechanisms determine the time window for coincidence detection. Direct electrical stimulation of the synaptic inputs in bird slices have shown this window is quite narrow, with a symmetry around zero delay, and with excitatory postsynaptic potentials (EPSPs) with similar kinetics for contralateral and ipsilateral synaptic inputs [28]. In the gerbil, Jercog et al. report differences in the synaptic response kinetics between contralateral versus ipsilateral postsynaptic potentials. With asymmetries in the rise times of the inputs, spiking is biased in favor of bilateral stimulation in which the faster EPSP leads, in this case the ipsilateral EPSP. This ipsilateral bias almost precisely counteracts the intrinsic axonal delay, such that activating the pathways simultaneously (equivalent to a zero-delay external ITD) leads to greatest firing. A model by Jercog et al. suggests the effect is crucially dependent on G_KLT_, on its amplitude and activation dynamics. The longer the initial rise of the compound synaptic potential, the more time G_KLT_ has to activate, the larger the conductance will be at the time of the peak in the EPSP, suppressing the voltage response, and reducing the likelihood of firing an action potential.

Thus, although the source(s) of the asymmetry in medial superior olive inputs remains open to debate, one major point emerges from the Jercog et al. study, which is that asymmetry in bilateral EPSP shapes could greatly influence coincidence detection and neural codes for ITD. In vivo, this asymmetry could come from almost anywhere—variation in the phase locking of the inputs, variation in the best frequency of the inputs, variation in axonal properties of the inputs, changes in inhibitory synaptic inputs, and differential expression of I_KLT_ in opposite dendrites. Certainly, the interplay of excitation and inhibition can shift ITD tuning curves. In the final analysis, biophysical examination of coincidence detection offers an outstanding opportunity to ask precise questions about neural coding.

## Abbreviations

EPSP: excitatory postsynaptic potential
G_KLT_: low-threshold potassium conductance
ITD: interaural time difference
